# New Therapeutic Strategies for Systemic Sclerosis—a Critical Analysis of the Literature

**DOI:** 10.1080/17402520500233437

**Published:** 2005-09

**Authors:** Gisele Zandman-Goddard, Nurit Tweezer-Zaks, Yehuda Shoenfeld

**Affiliations:** 1 Department of Medicine B and Center for Autoimmune Diseases Sheba Medical Center Sackler Faculty of Medicine Tel-Aviv University Tel-Hashomer Israel; 2 Department of Medicine F and the Rheumatology Unit Sheba Medical Center Sackler Faculty of Medicine Tel-Aviv University Tel-Hashomer Israel

## Abstract

Systemic sclerosis (SSc) is a multi-system disease characterized by skin fibrosis and visceral disease. Therapy is organ and pathogenesis targeted. In this review, we describe novel strategies in the treatment of SSc. Utilizing the MEDLINE and the COCHRANE REGISTRY, we identified open trials, controlled trials, for treatment of SSc from 1999 to April 2005. We used the terms scleroderma, systemic sclerosis, Raynaud's phenomenon, pulmonary hypertension, methotrexate, cyclosporin, tacrolimus, relaxin, low-dose penicillamine, IVIg, calcium channel blockers, losartan, prazocin, iloprost, N-acetylcysteine, bosentan, cyclophosphamide, lung transplantation, ACE inhibitors, anti-thymocyte globulin, and stem cell transplantation. Anecdotal reports were omitted.

Methotrexate, cyclosporin, tacrolimus, relaxin, low-dose penicillamine, and IVIg may be beneficial in improving the skin tightness in SSc. Calcium channel blockers, the angiotensin II receptor type 1 antagonist losartan, prazocin, the prostacyclin analogue iloprost, N-acetylcysteine and the dual endothelin-receptor antagonist bosentan may be beneficial for Raynaud's phenomenon. Epoprostenol and bosentan are approved for therapy of pulmonary hypertension (PAH). Other options under investigation include intravenous or aerolized iloprost. Cyclophosphamide (CYC) pulse therapy is effective in suppressing active alveolitis. Stem cell and lung transplantation is a viable option for carefully selected patients. Renal crisis can be effectively managed when hypertension is aggressively controlled with angiotensin converting enzyme (ACE) inhibitors. Patients should continue taking ACE inhibitors even after beginning dialysis in hope of discontinuing dialysis. Anti-thymocyte globulin and mycophenolate mofetil appear safe in SSc. The improvement in skin score and the apparent stability of systemic disease during the treatment period suggest that controlled studies of these agents are justified. Stem cell transplantation is under investigation for severe disease. Novel therapies are currently being tested in the treatment of SSc and have the potential of modifying the disease process and overall clinical outcome. The evaluation of these studies is still a difficult process.


*Clinical & Developmental Immunology*, September 2005; 12(3): 165–173

Taylor & Francis

Taylor & Francis Group

### New therapeutic strategies for systemic sclerosis—a critical analysis of the literature

GISELE ZANDMAN-GODDARD[1], NURIT TWEEZER-ZAKS[2], & YEHUDA SHOENFELD[1]

[1]*Department of Medicine B and Center for Autoimmune Diseases, Sheba Medical Center, Tel-Hashomer; Sackler Faculty of Medicine, Tel-Aviv University, Israel,* and [2]*Department of Medicine F and the Rheumatology Unit, Sheba Medical Center, Tel-Hashomer; Sackler Faculty of Medicine, Tel-Aviv University, Israel*

**Abstract**

Systemic sclerosis (SSc) is a multi-system disease characterized by skin fibrosis and visceral disease. Therapy is organ and pathogenesis targeted. In this review, we describe novel strategies in the treatment of SSc. Utilizing the MEDLINE and the COCHRANE REGISTRY, we identified open trials, controlled trials, for treatment of SSc from 1999 to April 2005. We used the terms scleroderma, systemic sclerosis, Raynaud's phenomenon, pulmonary hypertension, methotrexate, cyclosporin, tacrolimus, relaxin, low-dose penicillamine, IVIg, calcium channel blockers, losartan, prazocin, iloprost, N-acetylcysteine, bosentan, cyclophosphamide, lung transplantation, ACE inhibitors, anti-thymocyte globulin, and stem cell transplantation. Anecdotal reports were omitted.

Methotrexate, cyclosporin, tacrolimus, relaxin, low-dose penicillamine, and IVIg may be beneficial in improving the skin tightness in SSc. Calcium channel blockers, the angiotensin II receptor type 1 antagonist losartan, prazocin, the prostacyclin analogue iloprost, N-acetylcysteine and the dual endothelin-receptor antagonist bosentan may be beneficial for Raynaud's phenomenon. Epoprostenol and bosentan are approved for therapy of pulmonary hypertension (PAH). Other options under investigation include intravenous or aerolized iloprost. Cyclophosphamide (CYC) pulse therapy is effective in suppressing active alveolitis. Stem cell and lung transplantation is a viable option for carefully selected patients. Renal crisis can be effectively managed when hypertension is aggressively controlled with angiotensin converting enzyme (ACE) inhibitors. Patients should continue taking ACE inhibitors even after beginning dialysis in hope of discontinuing dialysis. Anti-thymocyte globulin and mycophenolate mofetil appear safe in SSc. The improvement in skin score and the apparent stability of systemic disease during the treatment period suggest that controlled studies of these agents are justified. Stem cell transplantation is under investigation for severe disease. Novel therapies are currently being tested in the treatment of SSc and have the potential of modifying the disease process and overall clinical outcome. The evaluation of these studies is still a difficult process.

**Keywords:** *Systemic sclerosis, bosentan, epoprostenol, IVIg, ACE inhibitors, stem cell transplantation*

### Introduction

Systemic sclerosis (SSc) is a multi-system disease characterized by skin fibrosis and visceral disease. The pathophysiology includes vascular damage, fibroblast proliferation, collagen production and activation of the immune system. Clinical manifestations include thickening of the skin, Raynaud's phenomenon, pulmonary artery hypertension (PAH) and pulmonary fibrosis, renal disease and involvement of other visceral organs. There is no single medication for the constellation of manifestations, rather therapy is organ and pathogenesis targeted (Table I). In this review, we describe novel strategies in the treatment of SSc.

### Skin involvement and early diffuse disease

Early diffuse SSc has no proven treatment. The efficacy of methotrexate (MTX) in improving the skin and other disease parameters was evaluated in 71 patients with early diffuse SSc of $<$ 3 years' duration

Correspondence: Y. Shoenfeld, Department of Medicine B, Sheba Medical Center, Tel Hashomer 52621, Israel. Tel: 972 3 5302652. Fax: 972 3 5352855. E-mail: shoenfel@post.tau.il

ISSN 1740-2522 print/ISSN 1740-2530 online © 2005 Taylor & Francis
DOI: 10.1080/17402520500233437

Table I. New therapies for SSC by organ involvement.

| Organ involvement | Mechanism | Treatment | References | No. of patients | Response |
|---|---|---|---|---|---|
| Skin | Imunosuppression | Methotrexate | Pope et al. 2001 | 35 | Modest |
| | Imunosuppression | Cyclosporin/ Tacrolimus | Morton and Powell 2000 | 24 | Beneficial |
| | Decreases collagen formation, inhibits angiotensin II | Relaxin | Seibold et al. 2000 | 68 | Effective |
| | | D-penicillamine (low dose) | Clements et al. 1999 | 68 | Same as high dose |
| | Imunosuppression | IVIg | Levy et al. 2000, Amital et al. 2003, Levy et al. 2004 | 26 | Beneficial |
| Raynaud's phenomenon/ Pulmonary hypertension | Vasodilatation | Calcium channel blockers | Thompson et al. 2001 | 109 | Beneficial |
| | Angiotensin I receptor antagonist | Losartan | Dziadzio et al. 1999 | 13 | Beneficial |
| | Alpha-1 inhibitor | Prazocin | Pope et al. 2000 | 40 | Modest |
| | Vasodilation, inhibitor of platelet aggregation | Epoprostenol | Badesch et al. 2000, Sitbon et al. 2002 | 56 | Beneficial |
| | Vasodilation | Iloprost | Pope et al. 2000, Olschewski et al. 2000 | 332 | IV beneficial |
| | Vascular | Treprostinil | Oudiz et al. 2004 | 90 | Beneficial |
| | Vascular | Beraprost | Matsukawa et al. 2002, Badesch et al. 2004 | 17 | Limited effect |
| | Endothelin-1 antagonist | Bosertan | Channick et al. 2001, Rubin et al. 2002, Sitbon et al. 2003, McLaughlin et al. 2005 | 32 | Beneficial |
| | Phosphodiesterase type 5 inhibition | Sildaefil | Lee et al. 2005 | 26 | Beneficial |
| | Vascular | N-acetyl cysteine | Sambo et al. 2001 | 22 | Safe |
| Fibrosing alveolitis | Immunosuppression | Cyclophosphamide | Davas et al. 1999, Griffiths et al. 2002, Giacomelli et al. 2002, Pakas et al. 2002, D'Angelo et al. 2003 | 16 | Beneficial |
| | Transplant | Lung transplant | Rosas et al. 2000 | | Limited effect |
| Gastrointestinal involvement | Enhances GI motility | Octreotide | Perlemuter et al. 1999 | 3 | Beneficial |
| Renal crisis | Vasodilation | ACE inhibitors | Steen and Medsger 2000 | 145 | Beneficial |
| Severe disease | Immunosuppression | ATG & MMF* | Stratton et al. 2001 | 13 | Investigational |
| | Immunosuppression | Stem cell transplant | Farge et al. 2004, Farge et al. 2005 | 41 | Good |

that were enrolled in a multicenter, randomized, placebo-controlled, double-blind trial (Pope et al. 2001). Thirty-five patients were treated with MTX (the maximum dose was 15 mg/week orally) and 36 with placebo for 12 months. At baseline, there were no statistically significant differences in skin scores, carbon monoxide diffusing capacity (DLCO), physician global assessment, or other secondary outcome measurements between the 2 treatment groups. At study completion, results slightly favored the MTX group when compared to placebo for the modified Rodnan skin score ($p < 0.17$) and for DLCO ($p < 0.2$). In addition, physician global assessment results favored MTX ($P < 0.035$), whereas patient global assessment did not differ significantly between groups. When between-group differences for changes in scores from baseline to 12 months were examined using intent-to-treat methodology, MTX appeared to have a favorable effect on skin scores ($p < 0.009$), but differences in the degree of change in the DLCO and physician global assessment were not significant. Although the results of this trial demonstrated a trend in favor of MTX versus placebo in the treatment of early diffuse SSc, the between-group differences were small and the power to rule out false-negative results was only 50%. These findings did not provide evidence that MTX is significantly effective in the treatment of early diffuse SSc.

Cyclosporin and tacrolimus are immunomodulatory drugs that act predominantly on T cells. Improvements in certain manifestations, particularly skin tightness, have been observed in a number of patients with SSc treated with these drugs. However, to date there have been no reports of their use in a routine clinical setting. In one study, patients attending clinical immunology clinics who had received cyclosporin and/or tacrolimus were identified. Details of their treatment, including drug dosage, duration and response to treatment, side effects and reasons for withdrawal, were recorded. Sixteen patients had been given cyclosporin and 13 of those had been treated for skin tightness. Half noticed a significant softening of their skin whilst on treatment, and resolution was observed in all four of the patients treated for digital vasculitis. Adverse effects were common and dose-limiting, and contributed to withdrawal in 12 out of 13 patients. Eight patients had been treated with tacrolimus; two of these had stopped the drug because of progression of their disease, one developed diarrhea, prompting withdrawal, one stopped tacrolimus following improvement, and four remained on the drug. Side effects had occurred in three patients. Improvements in skin occurred in approximately half of all cases of SSc treated with either cyclosporin or tacrolimus, suggesting a beneficial effect. Side effects, especially hypertension, are common with cyclosporin and often necessitate withdrawal. Adverse effects are also observed with tacrolimus, but in the small cohort so far treated, only one patient had stopped the drug for this reason (Morton and Powell 2000).

Relaxin is a pregnancy-related hormone that has tissue remodeling and antifibrotic effects. It inhibits endothelin-1, inhibits angiotensin II, and increases collagenase production. The efficacy, safety and dose-response effect of recombinant human relaxin in patients with SSc was assessed in a multicenter, parallel-group, randomized, double-blind, placebo-controlled trial (Seibold et al. 2000). Sixty-eight patients who had had stable, diffuse SSc (moderate to severe) for less than 5 years were enrolled. Recombinant human relaxin, 25 or 100 μg/kg of body weight per day, or placebo administered by continuous subcutaneous infusion over 24 weeks was administered. Modified Rodnan skin score was the primary efficacy measure. Secondary measurements were pulmonary function, the health assessment questionnaire (HAQ), and other measures of SSc that reflected fibrosis. Patients who received 25 μg/kg of recombinant human relaxin per day had significantly lower skin scores than those who received placebo ($p = 0.040$). Similar trends were noted in other outcome measures, including forced vital capacity, measures of oral aperture and hand extension, functional status, and global assessment. Patients who received 100 μg/kg of relaxin per day did not differ from those who received placebo. Drug-related adverse events included menometrorrhagia, reversible anemia, and complications of the subcutaneous drug administration system (site irritation and local infection). Following 24 weeks of recombinant human relaxin at a dose of 25 μg/kg/d, there was reduced skin thickening, improved mobility, and improved function in patients with moderate to severe disease.

The hypothesis that SSc patients taking high-dose D-penicillamine (D-Pen) would have a greater softening of skin, lower frequency of renal crisis, and better survival than patients taking low-dose D-Pen was tested in 134 SSc patients with early (≤18 months) diffuse cutaneous SSc in a 2-year, double-blind, randomized comparison of high-dose D-Pen (750–1000 mg/day) versus low-dose D-Pen (125 mg every other day) (Clements et al. 1999). All 134 patients were followed up for a mean ± SD of 4.0 ± 1.1 years to assess the frequencies of new-onset SSc renal crisis and mortality. The conclusion of the study was that the course of the skin score and the frequencies of SSc renal crisis and mortality in the high-dose D-Pen group were not different from those in the low-dose D-Pen group. Eighty percent of the adverse event-related withdrawals occurred in the high-dose D-Pen patients. Although this study did not answer the question of whether low-dose D-Pen is effective, it did suggest that there is no advantage to using D-Pen in doses higher than 125 every other day.

The response of SSc patients to treatment with intravenous immunoglobulin (IVIg) was demonstrated in 3 patients with progressive and rapidly deteriorating disease (mainly affecting the skin). The patients were planned to receive six monthly courses of high-dose IVIg (2 g/kg body weight over 5 days/course). All had a thorough physical examination, clinical evaluation by the modified Rodnan total skin thickness score, and measurement of the titers of PM-Scl antibodies before and after the treatment, and before and after each treatment course. Two of the three patients received six IVIg courses as planned and no adverse effects or disease progression occurred during the therapy. The third patient received three courses, after which he developed renal failure and later died of sepsis. All three patients had a significant decrease in their skin score after the treatment compared to that before the treatment. No modification of PM-Scl antibody titers was noted in any patient (Levy et al. 2000). IVIg may have a role in the treatment of SSc patients with rapidly deteriorating skin disease. The specific indications, as well as the safety of this treatment, should be further researched.

Eight patients with excess fibrotic reaction in the course of diverse diseases were analysed; a tendency that reverted with different IVIg treatment options. Three patients had SSc as their main feature. Fibrotic excess was reduced in all the patients by IVIg treatment. In five patients, the disease as a whole benefited from the infusion of immunoglobulins. IVIg may enhance resorption of fibrosis and promote healing in patients with fibrotic associated disorders (Amital et al. 2003). In another study, 15 SSc patients were treated with high dose IVIg (2 g/kg/course) for 3–6 monthly courses. Five patients had limited disease and 10 patients had diffuse disease. Efficacy was determined by a change in the modified Rodnan index and the HAQ quality of life score. The mean decrease in the skin score was significant ($p < 0.001$). A significant improvement in the patients' well-being and quality of life was reflected by a decrease in the HAQ score ($p = 0.03$) (Levy et al. 2004).

### Raynaud's phenomena/pulmonary hypertension

Most patients with SSc have Raynaud's phenomenon (RP), which is often more severe than idiopathic RP. A meta-analysis to determine the efficacy of calcium-channel blockers for the treatment of RP in SSc was performed (Thompson et al. 2001). Twenty-nine studies were found, of which 8 randomized controlled trials were eligible for inclusion. The total number of patients included was small ($n = 109$). Most trials included primary and secondary RP. The weighted mean difference of all calcium—channel blockers versus placebo (6 trials) and of nifedipine alone versus placebo (5 trials) for the reduction in the frequency of ischemic attacks over a 2-week period was −8.31 (95% confidence interval [95% CI] −15.71, −0.91) and −10.21 (95% CI −20.09, −0.34), respectively. The standardized mean difference of all calcium-channel blockers versus placebo (3 trials) and of nifedipine alone versus placebo (2 trials) for the reduction in the severity of ischemic attacks were −0.69 (95% CI −1.21, −0.17) and −0.99 (95% CI −1.74, −0.24), respectively. The conclusions were that calcium-channel blockers for RP in SSc have been tested in several small clinical trials and appear to lead to moderate clinical improvement in both the frequency and the severity of ischemic attacks. Further large, randomized controlled trial needs to be conducted.

The efficacy and tolerability of losartan, an angiotensin II receptor type 1 antagonist and nifedipine for the treatment of primary and secondary RP were compared in a pilot study (Dziadzio et al. 1999). In a randomized, parallel-group, controlled trial, 25 patients with primary RP or 27 RP patients secondary to SSc were allocated to receive 12 weeks' treatment with either losartan (50 mg/day) or nifedipine (40 mg/day). Primary outcome variables were the severity and frequency of RP episodes and findings on vascular measurements, including thermography and laser Doppler flowmetry. Serum levels of soluble adhesion molecules, endothelin 1, fibrinogen, von Willebrand factor, and procollagen type I N-terminal propeptide (PINP) were also measured. There was a reduction in the severity of RP episodes following treatment with losartan and with nifedipine, but this effect was greater in the losartan arm of the study ($P < 0.05$): Episode frequency was reduced only in the losartan group ($P < 0.01$ versus baseline). Symptomatic improvement was associated with a significant reduction in soluble vascular cell adhesion molecule 1 and PINP ($P < 0.01$). Subgroup analysis suggested that although these biochemical changes occurred mainly in SSc patients, the clinical benefit was greater in the primary RP group. This study confirmed the tolerability of short-term treatment of RP with losartan, and its clinical benefit. Further evaluation of this drug as a long-term treatment for SSc-associated RP should be considered, since it may have additional disease-modifying potential.

The effects and toxicity of prazosin, an alpha-1 inhibitor, compared to placebo proposed for the treatment of RP in SSc was evaluated (Thompson et al. 2001) Prazosin was found in two randomized controlled cross-over trials ($n = 40$) to be more effective than placebo in the treatment of RP secondary to SSc. However, the positive response was modest and side effects were not rare in those taking prazosin. Prazosin is modestly effective in the treatment of RP secondary to SSc.

Epoprostenol is a potent, short acting vasodilator and inhibitor of platelet aggregation produced by vascular endothelium. Epoprostenol is an effective vasodilator, reduces pulmonary resistance and platelet aggregation, increases cardiac output and demonstrates favorable effects on vascular remodulation.

Epoprostanol improved quality of life and survival in patients with PAH (Badesch et al. 2000, Sitbon et al. 2002).

Iloprost is a chemically stable synthetic analogue of prostacyclin (prostacyclin agonist) that decreases pulmonary vascular resistance. Iloprost is longer acting than epoprostenol with vasodilatory properties and platelet inhibitory effects. The effects and toxicity of prostacyclin analogues together with other agents proposed for the treatment of RP in SSc utilizing the Cochrane controlled trials register were analyzed (Pope et al. 2000). All randomized controlled trials comparing prostaglandin analogues versus placebo were eligible if they reported clinical outcomes within the start of therapy. Seven randomized trials and 332 patients were included. Five trials compared intravenous iloprost and one trial studied oral iloprost and oral cisaprost. In some trials, various doses of iloprost were used. Due to different efficacies of intravenous iloprost, oral iloprost and oral cisaprost, the overall efficacy of these drugs was somewhat diluted. Intravenous iloprost is effective in the treatment of RP secondary to SSc at decreasing the frequency and severity of attacks and preventing or healing digital ulcers. The effect seems to be prolonged after the intravenous infusion is given. Oral iloprost may have a lower efficacy than intravenous iloprost. However, cisaprost has minimal or no efficacy when given orally for the treatment of RP secondary to SSc.

Inhaled aerosolized iloprost, a stable prostacyclin analogue, has been considered a selective pulmonary vasodilator in the management of PAH (Olschewski et al 2000). One study assessed the efficacy of inhaled iloprost in the treatment of life-threatening PAH in an open, uncontrolled, multi-center study in intensive care units and PAH clinics at six university hospitals in Germany. Nineteen patients who had progressive right-heart failure despite receiving maximum conventional therapy (12 with primary PAH, 3 with PAH related to collagen vascular disease without lung fibrosis, and 4 with secondary PAH) were enrolled into the study. The patients were treated with inhaled iloprost, 6–12 times daily (50–200 μg/d). Right-heart catheterization and distance walked in 6 min at baseline and after 3 months of therapy were evaluated. During the first 3 months of therapy, New York Heart Association (NYHA) functional class improved in 8 patients and was unchanged in 7 patients. Four patients died, 3 of right-heart failure and 1 of sepsis. The acute hemodynamic response to inhaled iloprost was predominant pulmonary vasodilatation with little systemic effect at baseline and at 3 months (data available for 12 patients). Hemodynamic variables were improved at 3 months, and the distance walked in 6 min improved by 148 m ($p = 0.048$). Of the 15 patients who continued to use inhaled iloprost, 8 discontinued therapy; four had lung transplantation, 1 switched to intravenous prostacyclin therapy, and 3 died. Seven patients still received inhaled iloprost at 536 ± 309 days; at a mean dosage of 164 ± 38 μg/d. Inhaled iloprost may offer a new therapeutic option for improvement of hemodynamics and physical function in patients with life-threatening PAH and progressive right-heart failure that is refractory to conventional therapy.

Continuous subcutaneous infusion of treprostinil (initiated at 1.25 ng/kg/min, and titrated upward), a stable prostacyclin analogue was evauated for treating PAH in patients with connective tissue disease (CTD) in a randomized, double-blind, placebo-controlled, prospective trials in a subset of 90 patients with PAH and CTD, including systemic lupus erythematosus (SLE), diffuse and limited SSc, and mixed CTD/overlap syndrome. At baseline, most patients had NYHA class III symptoms. Continuous subcutaneous infusion of treprostinil in patients with PAH associated with CTD improved exercise capacity, symptoms of PAH, and hemodynamics (Oudiz et al. 2004).

Beraprost is the first orally active prostacyclin analogue. In the first of two randomized controlled trials, beraprost increased exercise capacity in patients with idiopathic PAH, with no significant changes in subjects with associated conditions. Hemodynamics did not change significantly, and no difference in survival was detected between the two treatment groups. The second study, showed that beraprost-treated patients had less disease progression at 6 months and confirmed the results of the previous trial. However, this improvement was no longer present at 9 or 12 months (Badesch et al. 2004).

The objective of another study was to assess the effect of beraprost sodium on pulmonary function in patients with SSc. Seventeen patients, with SSc and DLCO of less than 95%, received beraprost sodium for at least 12 months. DLCO levels in patients with SSc improved after the administration of beraprost sodium, probably due to the decrease in pulmonary vascular resistance accompanied by increased cardiac output (Matsukawa et al. 2002).

Endothelin 1 (ET1), a powerful endogenous vasoconstrictor and mitogen, might be a cause of pulmonary hypertension. ET1 causes vasoconstriction as well as endothelial activation, cell proliferation and fibrosis. The dual endothelin receptor antagonist bosentan is the first oral therapy approved for the treatment of pulmonary hypertension. Among patients with primary PAH or PAH related to SSc, randomized, double blind, placebo-controlled studies have demonstrated that bosentan therapy (62.5–250 mg twice daily) improves hemodynamics, exercise capacity and functional class (Channick et al. 2001, Rubin et al. 2002). In addition, a delay in time to clinical worsening was demonstrated in bosentan-treated patients compared with placebo, with clinical benefits maintained for up to 28 weeks (Rubin et al. 2002).

In a long-term, open-label extension study, the improvement in functional class persisted for up to one year (Sitbon et al. 2003). A recent study carries these results even further by administering bosentan as a first-line therapy, as well as evaluating long-term survival. In a long term, observational manner, first-line therapy with bosentan (125–250 mg daily), followed by other therapy if needed, improved survival in patients with primary PAH. The survival at two years was 89% compared to the predicted survival of 57%. Those patients with more advanced symptoms (functional class IV), and lower exercise tolerance had a worse outcome (McLaughlin et al. 2005). This last study does not refer to patients with secondary PAH, but there are previous studies showing similar results to treatment with bosentan among patients with primary or secondary PAH. The long term survival of SSc patients with PAH treated with bosentan is yet to be determined.

Phosphodiesterase type 5 (PDE5) inhibition has been proposed for the treatment for PAH. One study compared adding sildenafil, a PDE5 inhibitor to bosentan. Twenty-six patients with PAH, idiopathic or associated with CTD, functional class III, were randomized in a double-blind fashion to receive sildenafil (50 mg twice daily for 4 weeks, then 50 mg three times daily) or bosentan (62.5 mg twice daily for 4 weeks, then 125 mg twice daily) over 16 weeks. Measurements of efficacy included changes in right ventricular (RV) mass, 6-min walk distance, cardiac function, brain natriuretic peptide, and Borg dyspnea index. When analyzed by intention to treat, there were no significant differences between the two treatment groups. One patient on sildenafil died suddenly. Sildenafil added to conventional treatment reduces RV mass and improves cardiac function and exercise capacity in patients with severe PAH. Safety monitoring is important until more experience is obtained (Lee et al. 2005).

To date, 8 hemodynamic studies and 12 clinical trials (1 retrospective, 3 double-blind, 8 open-label) were performed to assess the efficacy of sildenafil for PAH (Wilkins et al. 2005). Sildenafil reduced PAH and pulmonary vascular resistance/peripheral vascular resistance index and tended to increase cardiac output/cardiac index compared with baseline. Sildenafil was comparable to nitric oxide and at least as effective as iloprost or epoprostenol in terms of its pulmonary vasoreactivity. Combination therapy with iloprost, nitric oxide, or epoprostenol resulted in enhanced and prolonged pulmonary vascular effects. Clinical trials suggest that sildenafil improves exercise tolerance and NYHA functional class, but large, randomized controlled trials are needed to confirm these findings. Overall, sildenafil was well tolerated.

The efficacy and tolerability of N-acetylcysteine (NAC) in patients with RP secondary to SSc was assessed in 22 patients in a multicenter, open clinical trial lasting 11 weeks and conducted in winter (Sambo et al. 2001). Primary outcome measures were frequency and severity of RP attacks, and number of digital ulcers. Secondary outcome measure was improvement in digital cold challenge test assessed by photoelectric plethysmography. Patients received a continuous 5 day intravenous infusion of NAC starting with a 2 h loading dose of 150 mg/kg subsequently adjusted to 15 mg/kg/h. All 22 patients completed the five-day infusion and 20 of them the post-treatment follow-up. Both frequency and severity of RP attacks decreased significantly compared to pretreatment values. Active ulcers were significantly less numerous at all follow-up visits. The mean recovery time improved measured by the cold challenge test. Side effects were minor, easily controlled, and reversible. N-acetylcysteine appears to be safe for the treatment of RP secondary to SSc and the preliminary data warrant further controlled studies.

### Fibrosing alveolitis/pulmonary fibrosis

Pulmonary fibrosis and PAH have become the most common causes of death in SSc. Consequently, the early diagnosis and treatment of pulmonary fibrosis is essential to improve morbidity and mortality in SSc patients. The prompt diagnosis of fibrosing alveolitis is imperative. There are some trials on the combination of cyclophosphamide (CYC) (IV or oral) and prednisone. In one study of 16 SSc patients with alveolitis, 8 patients were treated with monthly IV CYC pulse therapy (750 mg/m$^2$) for 12 months; the other 8 patients were treated with oral CYC (2–2.5 mg/kg/day) for the same period. All patients received concurrently prednisone (10 mg/day). Pulmonary function tests and high-resolution lung CT (HRCT) scans were performed before therapy and at 6 and 12 months. The results indicated that intravenous CYC pulse therapy was effective in suppressing active alveolitis. Although in this study it was not possible to compare pulse therapy with oral therapy because of the different pattern seen on high-resolution lung CT between the two groups, it seems that oral therapy was also effective in suppressing active alveolitis. Neither regimen improved pulmonary involvement when the reticular appearance indicating fibrosis predominated over the ground glass appearance on high resolution lung CT. Hence, it was concluded that either pulse or oral CYC therapy may improve the outcome of SSc patients with active alveolitis (Davas et al. 1999).

In another study, 14 consecutive SSc patients with lung involvement were treated with 6 pulses of IV methylprednisolone (10 mg/kg) and IV CYC (15 mg/kg) given at 3–4 weekly intervals and HRCT scans and lung function tests were performed at baseline and after the 6th pulse to assess the efficacy of treatment. Modified Rodnan skin scores improved significantly by 35%. HRCT scan scores improved significantly. Twelve of 13 patients experienced either improvement or stabilization of the HRCT score. Median DLCO and lung volumes remained stable during the first 12 months. However, after a median follow-up of 26 months, 67% of patients experienced deterioration in DLCO. The treatment was safe and well tolerated. While the IV regimen stabilized lung disease, deterioration in lung function occurred in the majority of patients upon discontinuation of therapy. The rate of deterioration of DLCO may be a useful marker for determining the intensity of treatment (Griffiths et al. 2002). Another study on 23 patients showed limited efficacy of IV CYC and prednisone for alveolitis. All patients received IV CYC (1000 mg/m$^2$ of body surface monthly for 6 months) and oral prednisone (25 mg daily for the first month and subsequently 5 mg daily of maintenance dosage for the remaining 5 months). At the end of the study, 8 patients were stable and 5 patients had a diffusion of the ground-glass to other segments (Giacomelli et al. 2002).

The combination of IV CYC with high dose prednisone (1 mg/kg/day for 4 weeks, then reducing the prednisone by 5 mg/day on alternating days each 2 weeks) seems to be more efficacious than with low dose prednisone ($<$ 10 mg/day) in an open label study in 28 SSc patients with fibrosing alveolitis. In the high dose steroid group, at 12 months there was significant improvement in the percentage of "ground glass" parenchymal lung involvement, as well as in the percentage of predicted FVC, the percentage of predicted DLCO, the percentage of skin involvement, and the severity of dyspnea. Substantial improvement was seen as early as 6 months (Pakas et al. 2002). Low-dose IV CYC has been shown to be efficacious in severe SLE with renal involvement (Houssiau et al. 2002). The aim of one trial was to test the safety of low-dose IV CYC in 8 patients with SSc. The patients were studied at baseline and after 6 months' intravenous CYC treatment (500 mg pulses at 2–4 week intervals). The therapy was well tolerated overall. No patient discontinued treatment because of side effects. Leukopenia, premature ovarian failure, hemorrhagic cystitis, microscopic hematuria and liver toxicity were never detected. Further studies are needed to assess both the efficacy and the long-term safety of the low-dose protocol (D'Angelo et al. 2003).

Lung transplantation in nine patients with SSc related end-stage lung disease was performed (Rosas et al. 2000). Patient characteristics included 6 with limited and 3 with diffuse SSc (seven were females). Pulmonary fibrosis was present in 78% and pulmonary hypertension in 44%. When compared to a similar group of transplant patients with non-scleroderma lung disease (primary pulmonary fibrosis), there was no significant difference in post-transplant survival at four years, mean annual incidence rate for acute rejection, and infection, or serum creatinine. Lung transplantation is a viable option for carefully selected patients with scleroderma related lung disease.

### Gastrointestinal involvement

Chronic intestinal pseudo-obstruction (CIPO) is a rare syndrome that may occur in association with scleroderma. Effective management is a major challenge. Treatment with subcutaneous octreotide in 3 patients was efficacious in the treatment of digestive symptoms in CIPO (Perlemuter et al. 1999). In 2 of the 3 cases, previous treatment with medications that enhance gastric motility including domperidone, cisapride, or erythromycin had been unsuccessful. All 3 patients underwent a regimen of oral antibiotics along with octreotide to stimulate small bowel motility. The effects of octreotide were evident within 48 h after the first injection in all patients. In 2 patients, the efficacy seemed to decrease after 1 week and 6 months respectively, but increasing the dosage led to another remission. CIPO in SSc is a severe condition that can evolve regardless of the underlying disease activity. Octreotide appears to be efficacious in improving both clinical symptoms and manometric patterns. When its therapeutic effect diminishes, increasing the dosage can be useful.

### Renal crisis

Although scleroderma renal crisis, a complication of SSc, can be treated with angiotensin-converting enzyme (ACE) inhibitors, its long-term outcomes are not known. In a prospective observational cohort study, 145 patients with SSc renal crisis who received ACE inhibitors and 662 patients with SSc who did not have renal crisis were evaluated (Steen and Medsger 2000). Among patients with renal crisis, the four outcomes studied were no dialysis, temporary dialysis, permanent dialysis and early death. Demographic, clinical, and laboratory data were compared to identify risk factors for specific outcomes. Follow-up was 5–10 years. Sixty-one percent of patients with renal crisis had good outcomes (55 received no dialysis, and 34 received temporary dialysis); only 4 of these (4%) progressed to chronic renal failure and permanent dialysis. More than half of the patients who initially required dialysis could discontinue it 3–18 months later. Survival of patients in the good outcome group was similar to that of patients with diffuse SSc who did not have renal crisis. Some patients (39%) had severe outcomes (permanent dialysis or early death). Renal crisis can be effectively managed when hypertension is aggressively controlled with ACE inhibitors. Patients should continue taking ACE inhibitors even after beginning dialysis in hopes of discontinuing dialysis.

### Diffuse visceral disease

The safety and efficacy of anti-thymocyte globulin (ATG) followed by mycophenolate mofetil (MMF) was assessed in a pilot study of 13 patients with recent-onset diffuse SSc (Stratton et al. 2001). Patients received ATG for 5 days, followed by MMF for 12 months. Adverse events, scleroderma skin score, hand contractures, scleroderma functional assessment, pulmonary function studies, echocardiogram and plasma creatinine concentration were recorded. Mean skin score decreased during the study ($P < 0.01$). Hand contractures worsened during the study. Mean measurements of systemic disease remained stable. One patient died after a scleroderma renal crisis. Five patients developed serum sickness after ATG treatment, but this was controlled by corticosteroid therapy. MMF therapy was well tolerated. ATG and MMF appear safe in SSc. The improvement in skin score and the apparent stability of systemic disease during the study period suggest that controlled studies of these agents are justified.

### Stem cell transplantation

Recent developments in haemopoietic stem cell transplantation (HSCT) permit the application of profound immunosuppression, followed by HSCT, or rescue, to autoimmune diseases such as SSc. The EBMT/EULAR report describes the longer outcome of patients originally described in addition to newly recruited cases. Only patients with SSc, treated by HSCT in European phase I–II studies from 1996 to 2002, with more than 6 months of follow up were included. Transplant regimens were according to the international consensus statements. Repeated evaluations analysed complete, partial, or non-response and the probability of disease progression and survival after HSCT. Among 57 patients aged 40 (9.1–68.7) years, the skin scores improved at 6 ($n = 37$ patients), 12 ($n = 30$), 24 ($n = 19$), and 36 ($n = 10$) months after HSCT ($p < 0.005$). After 22.9 (4.5–81.1) months, partial ($n = 32$) or complete response ($n = 14$) was seen in 92% and non-response in 8% of 50 observed cases and 35% of the patients with initial partial or complete response relapsed within 10 (2.2–48.7) months after HSCT. The transplant related mortality was 8.7%. Deaths related to progression accounted for 14% of the 23% total mortality rate. At 5 years, progression probability was 48% and the projected survival was 72%. This EBMT/EULAR report showed that response in two thirds of the patients after HSCT was durable with an acceptable transplant mortality rate. Based on these results, prospective, randomised trials are proceeding. (Farge et al. 2004). A recent study analyzed hematopoietic and immune reconstitution after autologous HSCT in 7 patients with SSc. B and T lymphocyte populations remained disturbed for at least 1 year after HSCT in SSc patients, which may reflect the persistence of an underlying disease mechanism (Farge et al. 2005).

In conclusion, SSc is a diversified disease with therapy targeted to a specific organ or mechanism of disease. The rarity of the disease precludes large control studies for evidence-based treatment. However, novel strategies are under investigation and may be an option for therapy.

### References

Amital H, Rewald E, Levy Y, Bar-Dayan Y, Manthorpe R, Engervall P, et al. 2003. Fibrosis regression induced by intravenous gammaglobulin treatment. Ann Rheum Dis 62:175–177.

Badesch DB, Tapson VF, McGoon MD, Brundage BH, Rubin LJ, Wigley FM, et al. 2000. Continuous intravenous epoprostenol for pulmonary hypertension due to the scleroderma spectrum of disease. A randomized, controlled trial. Ann Intern Med 21(132):425–434.

Badesch DB, McLaughlin VV, Delcroix M, Vizza CD, Olschewski H, Sitbon O, et al. 2004. Prostanoid therapy for pulmonary arterial hypertension. J Am Coll Cardiol 43(12 Suppl S):56S–61S.

Channick RN, Simonneau G, Sitbon O, Robbins IM, Frost A, Tapson VF, et al. 2001. Effects of the dual endothelin-receptor antagonist bosentan in patients with pulmonary hypertension: A randomised placebo-controlled study. Lancet 358: 1119–1123.

Clements PJ, Furst DE, Wong WK, Mayes M, White B, Wigley F, et al. 1999. High-dose versus low-dose D-penicillamine in early diffuse systemic sclerosis: Analysis of a two-year, double-blind, randomized, controlled clinical trial. Arthritis Rheum 42: 1194–1203.

D'Angelo S, Cuomo G, Paone C, Colutta E, La Montagna G, Valentini G. 2003. Low-dose intravenous cyclophosphamide in systemic sclerosis: A preliminary safety study. Clin Rheumatol 22:393–396.

Davas EM, Peppas C, Maragou M, Alvanou E, Hondros D, Dantis PC. 1999. Intravenous cyclophosphamide pulse therapy for the treatment of lung disease associated with scleroderma. Clin Rheumatol 18:455–461.

Dziadzio M, Denton CP, Smith R, Howell K, Blann A, Bowers E, et al. 1999. Losartan therapy for Raynaud's phenomenon and scleroderma: Clinical and biochemical findings in a fifteen-week, randomized, parallel-group, controlled trial. Arthritis Rheum 42:2646–2655.

Farge D, Passweg J, van Laar JM, Marjanovic Z, Besenthal C, Finke J, et al. 2004. EBMT/EULAR Registry. Autologous stem cell transplantation in the treatment of systemic sclerosis: Report from the EBMT/EULAR Registry. Ann Rheum Dis 63:974–981.

Farge D, Henegar C, Carmagnat M, Daneshpouy M, Marjanovic Z, Rabian C, et al. 2005. Analysis of immune reconstitution after autologous bone marrow transplantation in systemic sclerosis. Arthritis Rheum 52:1555–1563.

Giacomelli R, Valentini G, Salsano F, Cipriani P, Sambo P, Conforti ML, et al. 2002. Cyclophosphamide pulse regimen in the treatment of alveolitis in systemic sclerosis. J. Rheumatol 29:731–736.

Griffiths B, Miles S, Moss H, Robertson R, Veale D, Emery P. 2002. Systemic sclerosis and interstitial lung disease: A pilot study using pulse intravenous methylprednisolone and cyclophosphamide to assess the effect on high resolution computed tomography scan and lung function. J Rheumatol 29: 2371–2378.

Houssiau FA, Vasconcelos C, et al. 2002. D'Cruz D. Immunosuppressive therapy in lupus nephritis: The Euro-Lupus Nephritis Trial, a randomized trial of low-dose versus high-dose intravenous cyclophosphamide. Arthritis Rheum 46:2121–2131.

Lee AJ, Chiao TB, Tsang MP. 2005. Sildenafil for pulmonary hypertension. Ann Pharmacother 39:869–884.

Levy Y, Sherer Y, Langevitz P, Lorber M, Rotman P, Fabrizzi F, et al. 2000. Skin score decrease in systemic sclerosis patients treated with intravenous immunoglobulin–a preliminary report. Clin Rheumatol 19:207–211.

Levy Y, Amital H, Langevitz P, Nacci F, Righi A, Conforti L, et al. 2004. Intravenous immunoglobulin modulates cutaneous involvement and reduces skin fibrosis in systemic sclerosis: An open-label study. Arthritis Rheum 50:1005–1007.

Matsukawa Y, Saito O, Aoki M, Abe M, Nishinarita S, Sawada S, et al. 2002. Long-term administration of beraprost, an oral prostacyclin analogue, improves pulmonary diffusion capacity in patients with systemic sclerosis. Prostaglandins Leukot Essent Fatty Acids 67:45–49.

McLaughlin VV, Sitbon O, Badesch DB, Barst RJ, Black C, Galie N, et al. 2005. Survival with first-line bosentan in patients with primary pulmonary hypertension. Eur Respir J 25:244–249.

Morton SJ, Powell RJ. 2000. Cyclosporin and tacrolimus: Their use in a routine clinical setting for scleroderma. Rheumatology 39:865–869.

Olschewski H, Ghofrani HA, Schmehl T, Winkler J, Wilkens H, Hoper MM, et al. 2000. Inhaled Iloprost to treat severe pulmonary hypertension. An uncontrolled trial. German PPH Study Group. Ann Intern Med 132:435–443.

Oudiz RJ, Schilz RJ, Barst RJ, Galie N, Rich S, Rubin LJ, et al. 2004. Treprostinil, a prostacyclin analogue, in pulmonary arterial hypertension associated with connective tissue disease. Chest 126:420–427.

Pakas I, Ioannidis JP, Malagari K, Skopouli FN, Moutsopoulos HM, Vlachoyiannopoulos PG. 2002. Cyclophosphamide with low or high dose prednisolone for systemic sclerosis lung disease. J Rheumatol 29:298–304.

Perlemuter G, Cacoub P, Chaussade S, Wechsler B, Couturier D, Piette JC. 1999. Octreotide treatment of chronic intestinal pseudoobstruction secondary to connective tissue diseases. Arthritis Rheum 42:1545–1549.

Pope J, Fenlon D, Thompson A, Shea B, Furst D, Wells G, et al. 2000. Prazosin for Raynaud's phenomenon in progressive systemic sclerosis. Cochrane Database Syst Rev 2: CD000956.

Pope J, Fenlon D, Thompson A, Shea B, Furst D, Wells G, et al. 2000. Iloprost and cisaprost for Raynaud's phenomenon in progressive systemic sclerosis. Cochrane Database Syst.

Pope JE, Bellamy N, Seibold JR, Baron M, Ellman M, Carette S, et al. 2001. A randomized, controlled trial of methotrexate versus placebo in early diffuse scleroderma. Arthritis Rheum 44:1351–1358.

Rosas V, Conte JV, Yang SC, Gaine SP, Borja M, Wigley FM, et al. 2000. Lung transplantation and systemic sclerosis. Ann Transplant 5:38–43.

Rubin LJ, Badesch DB, Barst RJ, Galie N, Black CM, Keogh A, et al. 2002. Bosentan therapy for pulmonary arterial hypertension. N Engl J Med 346:1258.

Sambo P, Amico D, Giacomelli R, Matucci-Cerinic M, Salsano F, Valentini G, et al. 2001. Intravenous N-acetylcysteine for treatment of Raynaud's phenomenon secondary to systemic sclerosis: A pilot study. J Rheumatol 28:2257–2262.

Seibold JR, Korn JH, Simms R, Clements PJ, Moreland LW, Mayes MD, et al. 2000. Recombinant human relaxin in the treatment of scleroderma. A randomized, double-blind, placebo-controlled trial. Ann Intern Med 132:871–879.

Sitbon O, Humbert M, et al. 2002. Nuner H.Intravenous infusion of epoprostanol in severe pulmonary hypertension: Long term survival and prognostic factors. J Am Coll Cardiol 40:770–778.

Sitbon O, Badesch DB, Channick RN, et al. 2003. Effects of the dual endothelin receptor antagonist bosentan in patients with pulmonary artery hypertension. A 1-year follow-up study. Chest 124:247–254.

Steen VD, Medsger, TA. Jr. 2000. Long-term outcomes of scleroderma renal crisis. Ann Intern Med 133:600–603.

Stratton RJ, Wilson H, Black CM. 2001. Pilot study of anti-thymocyte globulin plus mycophenolate mofetil in recent-onset diffuse scleroderma. Rheumatology (Oxford) 40:84–88.

Thompson AE, Shea B, Welch V, Fenlon D, Pope JE. 2001. Calcium-channel blockers for Raynaud's phenomenon in systemic sclerosis. Arthritis Rheum 44:1841–1847.

Wilkins MR, Paul GA, Strange JW, Tunariu N, Gin-Sing W, Banya WA, et al. 2005. Sildenafil versus Endothelin Receptor Antagonist for Pulmonary Hypertension (SERAPH) Study. Am J Respir Crit Care Med 171:1292.

